# Case Report: Deep Cerebellar Stimulation for Tremor and Dystonia

**DOI:** 10.3389/fneur.2021.642904

**Published:** 2021-03-05

**Authors:** Shiro Horisawa, Kotaro Kohara, Taku Nonaka, Tatsuki Mochizuki, Takakazu Kawamata, Takaomi Taira

**Affiliations:** Department of Neurosurgery, Neurological Institute, Tokyo Women's Medical University, Tokyo, Japan

**Keywords:** cerebellum, cerebellar stimulation, dentate nucleus, superior cerebellar peduncle, tremor, dystonia

## Abstract

**Background:** The cerebellum plays an important role in the pathogenesis and pathophysiology of movement disorders, including tremor and dystonia. To date, there have been few reports on deep cerebellar stimulation.

**Case Report:** The patient was a 35-year-old previously healthy man with no history of movement disorders. He developed a tremor and stiffness in his left hand at the age of 27 years, which was diagnosed as a dystonic tremor. We performed right thalamotomy, which resulted in a complete resolution of the tremor; however, the dystonia persisted. Subsequently, the patient developed left foot dystonia with inversion and a newly developed tremor in the right hand and foot. The patient underwent left ventralis intermedius (VIM) deep brain stimulation (VIM-DBS) and left pallidothalamic tract DBS (PTT-DBS). Left VIM-DBS completely resolved the right hand and foot tremor, and PTT-DBS significantly improved the left hand and foot dystonia. Three months postoperatively, the patient developed an infection and wound disruption at the surgical site. We performed palliative surgery for deep cerebellar stimulation via the posterior cranial region, which was not infected. The surgery was performed under general anesthesia with the patient lying in the prone position. Eight contact DBS electrodes were used. The placement of electrodes extended from the superior cerebellar peduncle to the dentate nucleus. Both the right hand and foot tremor improved with right cerebellar stimulation. Further, both the left hand and foot dystonia improved with left cerebellar stimulation. Right and left cerebellar stimulation led to no improvement in the left hand and foot dystonia and right hand and foot tremor, respectively. Stimulation-induced complications observed in the patient included dizziness, dysphagia, and dysarthria. After the surgery, the patient developed hypersalivation and hyperhidrosis in the left side of the body, both of which did not improve with adjustments of stimulation parameters. At the 6-month follow-up, the tremor and dystonia had almost completely resolved.

**Conclusion:** Deep cerebellar stimulation deserves consideration as a potential treatment for tremor and dystonia.

## Introduction

The cerebellum plays an important role in the pathogenesis and pathophysiology of movement disorders, including tremor and dystonia (1–4). The dentate nucleus is the largest deep cerebellar nucleus and the main output site of the cerebellum. During the 1960–1970's, dystonia and cerebral palsy were treated by ablation or stimulation of the dentate nucleus (5, 6). Cooper et al. reported significant improvements in spasticity, gait, and speech impairment in cerebral palsy after chronic cerebellar stimulation (5). Lesioning of dentate nucleus (dentatotomy) was reported to reduce ipsilateral muscle tone in cerebral palsy and dystonia (6, 7). However, this procedure was abandoned for unknown reasons. To date, there have been few reports on deep cerebellar stimulation (8–11). Sokal et al. reported the treatment of secondary dystonia by anterior cerebellar DBS, which resulted in a 42.8% improvement according to the Unified Dystonia Rating Scale (10). We also reported a single case of generalized fixed dystonia treated by superior cerebellar peduncle DBS with a 39.3% improvement according to Barry-Albright dystonia rating scale (8). Brown et al. reported deep brain stimulation (DBS) of the bilateral superior cerebellar peduncle for acquired hemidystonia with 38.6% improvement according to Burke-Fahn-Marsden dystonia rating scale-movement score (BFMDRS-MS) 6 months post-operatively (9). Cury et al. reported DBS of the dentate nucleus for post-stroke tremor with 50% improvement according to Fahn-Tolosa-Marin (FTM) score (7). Current application of deep cerebellar stimulation is limited to secondary movement disorders. We report a single case of dystonia and tremor without an antecedent pathology, which was successfully treated with bilateral deep cerebellar stimulation.

## Case Description

The patient was a 35-year-old previously healthy man with no history of movement disorders. He developed a tremor and stiffness in his left hand at the age of 27 years. Head MRI revealed no abnormality. Dopamine transporter single photon emission computerized tomography was also normal, and he was diagnosed with dystonic tremor. Several oral medications including trihexyphenidyl (6 mg), clonazepam (3 mg), propranolol (120 mg), primidone (750 mg), and L-dopa (300 mg) proved ineffective. Consequently, he was referred to our hospital for surgical intervention. Upon clinical examination, resting, postural and intention tremor were confirmed in the patient's left hand. The patient also felt left hand stiffness, which caused slight difficulty in opening his left hand. No other tremor and dystonia were confirmed in other body parts. There was no rigidity, bradykinesia, walking disturbance, or balance disorder. Alcohol intake did not improve the symptoms. There was no significant abnormality in genetic testing for dystonia and Parkinson's disease. Considering these clinical findings, the probable diagnosis was dystonic tremor. His FTM tremor rating scale (score: 0–144; higher scores indicate more severe tremor) score was 53.

We performed right ventralis intermedius (VIM) thalamotomy, which resulted in a complete resolution (FTM 0) of the tremor. Two years later, he developed a left-hand tremor with clenched fist (dystonic posture), which is suggestive of dystonic tremor (Supplementary Video 1). We performed right VIM-ventro-oralis (VIM-VO) thalamotomy. This procedure stopped the tremor; however, the dystonic posture persisted. One month later, the patient developed left foot dystonia with inversion and a newly developed tremor in the right hand and foot. The BFMDRS-MS (score: 0–120; higher scores indicate more severe dystonia) was 12 based on the left hand and left foot, each (Supplementary Video 2). Three months after the previous procedure, we performed left VIM-DBS and left pallidothalamic tract-DBS (PTT-DBS) (Boston Scientific, Vercise Cartesia™ Directional Lead, Vercise Gevia™ DBS System, Valencia, CA, USA) in the patient. Left VIM-DBS at 100 μs/100 Hz/2.5 mA completely resolved the right hand and foot tremor, and PTT-DBS at 100 μs/100 Hz/3.0 mA significantly improved the left hand and foot dystonia (BFMDRS-MS: left hand, 2; left foot, 1) (Supplementary Video 2). Stimulation-induced dysarthria and parkinsonism (postural reflex disturbance, akinesia) were managed by adjusting the stimulation parameters. Three months post-operatively, the patient developed an infection and wound disruption at the surgical site. Despite long-term antibiotic administration, exchange of battery and extension cable in the implanted pulse generator (IPG), and repetitive saline washing, the infection and wound disruption gradually worsened, which warranted the requirement for removal of the entire DBS system. Due to the severity of the tremor and dystonia, switching off the DBS device resulted in the patient being unable to stand or walk. Therefore, after obtaining written informed consent from the patient and approval from the ethics committee at our hospital, we performed palliative surgery for deep cerebellar stimulation via the posterior cranial region, which was not infected.

**Figure 1:**
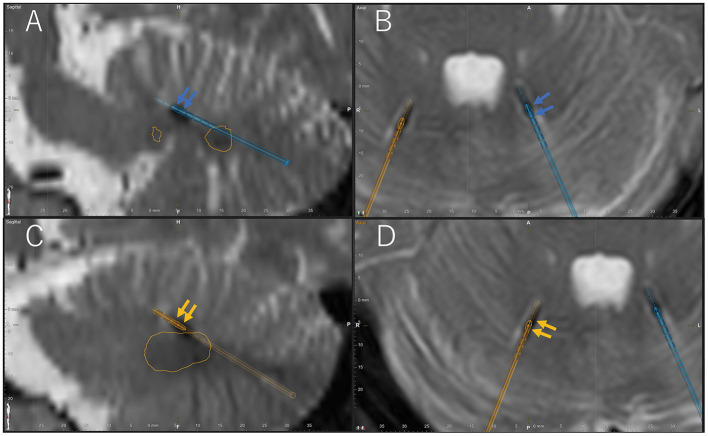
Location of active contacts of final stimulation settings confirmed by Brainlab Elements on the post-operative T2-weighted magnetic resonance imaging (MRI). Blue arrow indicates active cathode contacts of the left electrode (blue electrode) in the sagittal **(A)** and axial **(B)** postoperative T2-weighted MRI. Orange arrow indicates active cathode contacts of the right electrode (orange electrode) in the sagittal **(C)** and axial **(D)** postoperative T2-weighted MRI. Orange circle shows the bilateral dentate nucleus. A, anterior; P, posterior; H, head; F, foot; L, left; R, right.

The surgery was performed under general anesthesia with the patient lying in the prone position. Eight contact DBS electrodes (Boston Scientific, Vercise™ DBS Lead, Valencia, CA, USA) were used. The placement of electrodes extended from the superior cerebellar peduncle to the dentate nucleus (Figure 1). After electrode placement, the IPG (Boston Scientific, Vercise Gevia™ DBS System, Valencia, CA, USA) was placed in the left side of the chest. Subsequently, the infected DBS device was removed in the same surgery.

Both the right hand and foot tremor improved with right cerebellar stimulation (1-C+, 120 μs/130 Hz/0.5 mA, Figure 1A; and 8-C+, 120 μs, 130 Hz/2.5 mA, Figure 1B, respectively) (Supplementary Video 3). The FTM score was 0. Further, both the left hand and foot dystonia improved with left cerebellar stimulation (4-C+, 120 μs, 130 Hz, 2.0 mA, Figure 1C; and 6-C+, 120 μs, 130 Hz, 2.0 mA, Figure 1D, respectively) (Supplementary Video 3). The BFMDRS-MS for the left arm was 2 and for the left leg was 0. Right and left cerebellar stimulation led to no improvement in the left hand and foot dystonia and right hand and foot tremor, respectively.

**Figure 2:**
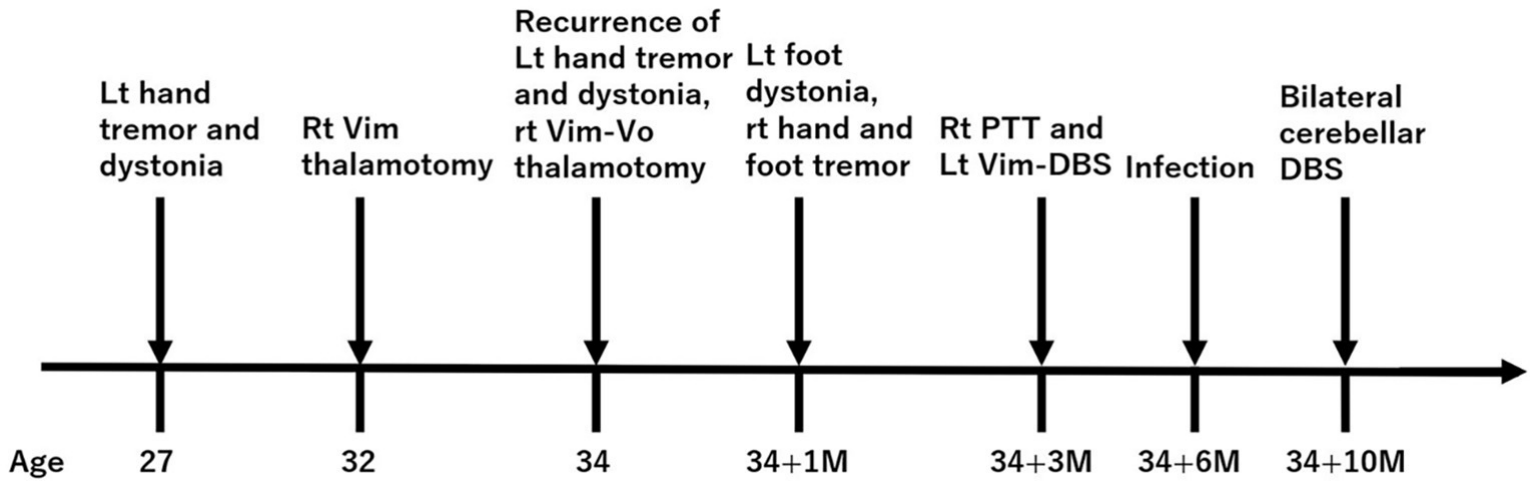
Time course of symptoms and interventions. Values indicate years. M, month.

Stimulation-induced complications observed in the patient included dysmetria, dizziness, dysphagia, and dysarthria, which were mild and adjustable. After the surgery, the patient developed hypersalivation and hyperhidrosis in the left side of the body, both of which did not improve with adjustments of stimulation parameters. At the 6-month follow-up, tremor and dystonia were almost completely resolved. The final stimulation settings were as follows: 3–4-C+, 300 μs, 104 Hz, 1.8 mA in the left IPG, and 11–12-C+, 300 μs, 104 Hz, 0.5 mA in the right IPG. The FTM score and BFMDRS-MS of the left arm and left leg at the 6-month follow-up were 0, 2, and 0 respectively. The time course of the symptoms and interventions is shown in Figure 2.

## Discussion

This case report presents two interesting findings. First, the effects of cerebellar DBS on tremor and dystonia were similar to those of VIM- and PTT-DBS. Second, upper and lower limb symptoms responded well to different stimulation contacts.

The present patient experienced recurrence of left hand tremor 2 years after the first VIM thalamotomy. Recurrence of tremor or dystonia by insufficient ablation or misalignment of the target after thalamotomy usually occurs within 3 months of surgery (12). Recurrence 2 years after thalamotomy suggests that this recurrence was due to disease progression. Although we performed VIM-VO thalamotomy, the patient still showed refractory left hand dystonia and newly developed left foot dystonia. We believed that additional lesioning surgery carries a higher risk of irreversible complications associated with bilateral thalamotomy such as dysarthria, dysphonia, and dysphagia. Therefore, we selected DBS as further surgical treatment. Although the majority of surgical treatment of dystonia is globus pallidus interna (GPi)-DBS, distal limb dystonia is frequently treated by VO thalamotomy (13–16). Fukaya et al. reported that GPi-DBS had less effect on focal hand dystonia than VO-DBS (17). The optimal stimulation site within GPi for focal hand dystonia remains unknown. We also frequently experienced a poor reaction of distal limb dystonia to pallidotomy or GPi-DBS. The pallidothalamic tract is a pallidal efferent fiber to motor thalamic nuclei including the VO nucleus (18). Our previous study suggested that ablation of the pallidothalamic tract was highly expected to be effective for dystonia (19). Most pallidothalamic fibers pass through Forel's field H1, within a diameter of <4 mm, which suggest that Forel's field H1 is a suitable target for DBS (20). Therefore, we selected PTT as a treatment target of DBS, and PTT-DBS almost completely improved the left hand and foot dystonia.

A possible therapeutic mechanism for cerebellar DBS is the neuromodulation of the cerebellar output pathway including the dentate-rubro-thalamic tract (DRTT). The DRTT conveys excitatory information from the dentate nucleus to the primary motor cortex. Neuromodulation of the DRTT controls cortical excitability, which may lead to improvement of tremor and dystonia (21–24). The dentate nucleus has a motor (dorsal side) and non-motor (ventral side) territory; the ventral dentate nucleus projects to the prefrontal cortex and contributes to cognitive function, whereas the dorsal dentate nucleus projects to the primary motor and supplementary motor cortex and contributes to motor function (25). Targets reported in previous studies include the dentate nucleus, superior cerebellar peduncle, and anterior cerebellum, all of which are responsible for the output structure of the cerebellum (8–11). The present case showed 100% improvement of FTM and 91.7% improvement of BFMDRS-MS. The difference between previous studies and the present case is due to the patient's background. Previous studies have provided cerebellar DBS for movement disorders with acquired tremor or dystonia, which are refractory to conventional DBS therapy, compared to idiopathic dystonia and tremor. The present patient had no abnormality on head MRI at the initial onset evaluation and no history of head trauma, stroke, or epilepsy, which suggest that the movement disorders in the present patient result from primary origin. Therefore, VIM-DBS and PTT-DBS were quite effective for tremor and dystonia in the present case. While the improvements achieved by cerebellar DBS were same as those achieved by VIM-DBS and PTT-DBS, complications associated with the procedures were completely different between cerebellar DBS and VIM-/PTT-DBS. Stimulation-induced complications associated with VIM-DBS and contralateral PTT-DBS were dysarthria, dysesthesia, and parkinsonism including postural reflex disturbance, akinesia, and disdiadochokinesia. In cerebellar DBS, dysmetria, dizziness, dysarthria, dysphagia, hypersalivation, and hyperhidrosis were confirmed.

Cerebellar stimulation-induced complications including ipsilateral leaning, dizziness, appendicular ataxia, gaze deviation, nausea, decreased verbal fluency, and forced laughing have been reported (9, 10, 26). Interestingly, the present patient showed hypersalivation and hyperhidrosis after the surgery, which have not been reported. Stimulation-induced hyperhidrosis has been reported in association with DBS of the subthalamic nucleus (STN) and posterior subthalamic area (PSA) (27–30). In reports of STN-DBS-induced hyperhidrosis, the active contacts were posterior-medial to the STN, superior-medial to the STN, or between the STN and red nucleus (27–29). Blomstedt reported that contacts causing hyperhidrosis in precisely located electrodes within the PSA were 11 mm lateral to the midline, 7.6 mm posterior to the midcommissural point, and 3.7 mm below the anterior commissure-posterior commissure line (30). Considering these findings, the area associated with stimulation-induced hyperhidrosis seems to be medially located to the STN, where the DRTT passes through. Stimulation to the cerebellar output structures (superior cerebellar peduncle or dentate nucleus) conveys electrical activation to the part of the DRTT located medial to the STN, which might result in the similar phenomenon of STN- or PSA-DSB inducing hyperhidrosis. Additionally, the connections between the cerebellum and the hypothalamus have been well-reported. Preoptic area of the anterior hypothalamus and dorsomedial hypothalamus are considered to play an important role for thermoregulation (31, 32). Both hypothalamic structures are connected with the cerebellum via the superior cerebellar peduncle (33). Cerebellar stimulation at the superior cerebellar peduncle might affect the hypothalamic structures associated with thermoregulation, which might lead to hyperhidrosis. Hypersalivation is reported in association with STN-, pallidal, and thalamic DBS. However, detailed mechanism of stimulation-induced hypersalivation remains unknown. The salivary nuclei within the medulla oblongata, which plays a central role in salivation, receive nerve projections including excitatory or inhibitory effect on salivary secretion from the cortex, and connects to the lateral hypothalamus (34). Hypothalamic-cerebellar and cerebellar-hypothalamic connections *via* the superior cerebellar peduncle also include lateral hypothalamus (33). Both hyperhidrosis and hypersalivation observed in this case suggest that cerebellar stimulation may have neuromodulation effects on hypothalamic function.

In conclusion, cerebellar stimulation deserves consideration as a potential treatment for tremor and dystonia.

## Data Availability Statement

The original contributions presented in the study are included in the article/Supplementary Material, further inquiries can be directed to the corresponding author/s.

## Ethics Statement

The studies involving human participants were reviewed and approved by Ethics committee of Tokyo Women's Medical University. The patients/participants provided their written informed consent to participate in this study.

## Author Contributions

SH: conception, organization, execution, and writing of the draft and figure. KK, TN, and TM: execution. TK: organization. TT: conception, organization, and execution. All authors contributed to the article and approved the submitted version.

## Conflict of Interest

The authors declare that the research was conducted in the absence of any commercial or financial relationships that could be construed as a potential conflict of interest.

## Abbreviations

FTM: Fahn-Tolosa-Marin tremor rating scale
GPi: globus pallidus interna
VIM: ventralis intermedius
VIM-VO: ventralis intermedius-ventro-oralis
BFMDRS-MS: Burke-Fahn-Marsden dystonia rating scale-movement score
VIM-DBS: ventralis intermedius-deep brain stimulation
PTT-DBS: pallidothalamic tract-deep brain stimulation
IPG: implanted pulse generator
DRTT: dentate-rubro-thalamic tract
STN: subthalamic nucleus
PSA: posterior subthalamic area.
